# A plasma lipid signature in acute human traumatic brain injury: Link with neuronal injury and inflammation markers

**DOI:** 10.1177/0271678X241276951

**Published:** 2024-08-26

**Authors:** Isabell Nessel, Luke Whiley, Simon C Dyall, Adina T Michael-Titus

**Affiliations:** 1 Centre for Neuroscience, Surgery and Trauma, Blizard Institute, Barts and The London School of Medicine and Dentistry, Queen Mary University of London, London, UK; 2 Health Futures Institute, 5673 Murdoch University, Murdoch, Australia; 3 School of Life and Health Sciences, University of Roehampton, London, UK

**Keywords:** Brain lipids, omega-3 fatty acids, lipidomics, traumatic brain injury, neurofilament light

## Abstract

Traumatic brain injury (TBI) leads to major membrane lipid breakdown. We investigated plasma lipids over 3 days post-TBI, to identify a signature of acute human TBI and assess its correlation with neuronal injury and inflammation. Plasma from patients with TBI (Abbreviated Injury Scale (AIS)3 - serious injury, n = 5; AIS4 - severe injury, n = 8), and controls (n = 13) was analysed for lipidomic profile, neurofilament light (NFL) and cytokines, and the omega-3 index was measured in red blood cells. A lipid signature separated TBI from controls, at 24 and 72 h. Major species driving the separation were: lysophosphatidylcholine (LPC), phosphatidylcholine (PC) and hexosylceramide (HexCer). Docosahexaenoic acid (DHA, 22:6) and LPC (0:0/22:6) decreased post-injury. NFL levels were increased at 24 and 72 h post-injury in AIS4 TBI vs. controls. Interleukin (IL-)6, IL-2 and IL-13 were elevated at 24 h in AIS4 patients vs. controls. NFL and IL-6 were negatively correlated with several lipids. The omega-3 index at admission was low in all patients (controls: 4.3 ± 1.1% and TBI: 4.0 ± 1.1%) and did not change significantly over 3 days post-injury. We have identified specific lipid changes, correlated with markers of injury and inflammation in acute TBI. These observations could inform future lipid-based therapeutic approaches.

## Introduction

Traumatic brain injury (TBI) is a condition with significant heterogeneity, which can lead to major lifelong consequences for patients.^1^ These include fatigue, headaches, impaired memory and executive function, reduced concentration and attention, impaired information processing capacity, depression and anxiety, irritability, emergence of aggression, sleep disturbances and sexual dysfunction. Its therapeutic management remains a major unmet need. The severity of TBI is graded acutely using clinical scores such as the Glasgow Coma Scale (GCS), based on motor, eye, and verbal responses, which reflect the patient’s level of consciousness. However, there is consensus that the GCS has major limitations, therefore there is ongoing research effort to define biomarkers which could be used in the injury acute phase, which would enable a better characterization and stratification of patients, both for outcome prediction and for the refinement of clinical trials. Much work on blood biomarkers has been focused on various protein biomarkers,^2–4^ which directly reflect the impact of the injury on the integrity of neuronal and non-neuronal cells, and which are detectable in the central and peripheral compartments. For example, neurofilament light chain (NFL), a neuronal cytoskeletal protein which is detected at high levels in the cerebrospinal fluid (CSF) and plasma after injury, is well established as an indicator of brain structural damage.^5,6^ NFL levels, as part of a protein biomarker panel measured within the first 5 days post-TBI, adds independent information which improves the prediction of injury outcome.^7^ Much less is known about whether specific lipids could also become sensitive and reproducible biomarkers of injury. The brain is enriched in lipids,^8^ and has the highest lipid content after adipose tissue. Numerous different types of lipids detected in the central nervous system (CNS) have significant structural and cellular signalling roles and are directly involved in fundamental neuronal processes such as neurogenesis, impulse conduction, synaptogenesis and synaptic transmission.^9^ A well-documented change after experimental or clinical TBI, is the alteration in brain tissue lipid content, involving various lipid species, such as fatty acids, phospholipids (PL), glycerolipids, sterols and sphingolipids.^10^ This leads to the question whether a lipid signature of acute human TBI could be identified in the peripheral circulation, which would reflect key events in the central compartment and give, very early after injury, an indication of the severity of injury and its evolution in the subacute period, thus providing potentially more insight into prediction of individual neurological outcomes. Experimental and clinical studies over the last decade have started addressing this question. Bahado-Singh et al.^11^ identified lipids as part of a complex serum metabolomic panel, in a closed TBI mouse model, and showed that lipids such as phosphatidylcholine (PC) 34:4 are part of a set that differentiates TBI from control animals, within the first 24 h post-injury. In moderate TBI in the rat, Hogan et al.^12^ identified within the first week post-injury, a lipid panel including free fatty acids (FFA), PL, diacylglycerols (DAG) and sphingolipids such as sphingomyelin (SM), which separated the brain injured animals from naive or sham-operated animals. More recently, Anyaegbu et al.^13^ identified lipid profiles in a rat model of repetitive mild TBI (mTBI), with a distinct lipid signature for 1x and 2x mTBI that included lysophospholipids (LysoPL), PL and FFA. Daley and colleagues^14^ identified in the plasma of young athletes a metabolomic panel at 2–3 days after concussion, which included several PC species that accounted for 28% of the variance. Later, in a study in college athletes by Fiandaca et al.,^15^ LysoPL were part of the distinct plasma metabolomic signature detected within 6 days after a mTBI, and Thomas and colleagues^16^ identified in injured patients a metabolic signature at 24 h post-TBI which included several choline-containing lipids.

In this study, we analysed peripheral lipid changes that occur in human plasma acutely post-TBI, from 2 h after injury and over the first 3 days, with a particular focus on PL, as they are key structural components of cellular membranes and also act as a source of fatty acids which can be released after the action of phospholipases. In particular, the activation of phospholipase A_2_ is a very early event after injury in the central nervous system.^17^ The subsequent release of fatty acids can be detected in the CSF, and the intensity of PL enzymatic hydrolysis is negatively correlated in human TBI with the neurological outcome.^18^ The released fatty acids include omega-3 long-chain polyunsaturated fatty acids (PUFA) such as docosahexaenoic acid (DHA, 22:6n-3), which has intrinsic signalling roles, important for neural development and normal brain function,^19^ and which is also a precursor to a range of lipid mediators involved in the resolution of inflammation, such as resolvins, protectins and maresins.^9,20^ Furthermore, DHA has been shown to have significant potential as a neuroprotective agent when administered acutely in experimental TBI.^21–23^

The aim of our study was to correlate the lipid changes in plasma in the first 3 days of hospital admission after a TBI, with changes in other specific parameters which reflect injury severity and are linked to outcome. The evolution of plasma lipid changes was correlated with the dynamics of the neuronal injury marker NFL^6^ and also the levels of several cytokines. TBI triggers a complex inflammatory response, characterised by increases in the levels of various cytokines and chemokines, with pro-inflammatory and anti-inflammatory activities, the balance of which controls the evolution and resolution of inflammation after injury and influences outcome,^24–26^ and we selected a few key cytokines for a first exploration of a potential correlation systemically with lipid changes. Pulliam and colleagues recently showed in a rat model of closed-head mTBI correlations in brain tissue between lipid changes post-injury and the levels of various cytokines.^27^

We also assessed the omega-3 PUFA status of patients by measuring the omega-3 index (O3I), which reflects the relative levels of the omega-3 long-chain fatty acids eicosapentaenoic acid (EPA, 20:5n-3) and DHA in red blood cells; this index has been associated with higher cognitive function^28,29^ and also with prediction of all-cause mortality.^30^

## Material and methods

### Patients

For this analysis, samples from patients from the Royal London Hospital were selected from the Activation of Coagulation and Inflammation in Trauma (ACIT)-2 trial (ISRCTN12962642). The ACIT-2 trial is an observational study investigating the systemic inflammatory, coagulation and genomic response in humans to severe injury and bleeding after major trauma. Since 2008, adult patients receiving either pre-hospital care from the London Air Ambulance or requiring full trauma team activation when arriving at the emergency department of the Royal London Hospital were recruited to the ACIT-2 trial within 2 h of their injury. Samples were originally collected under a waiver of consent, due to the emergency nature. Written informed consent from the patient or next of kin was obtained as soon after enrolment as possible. The study was approved by London–City & East Research Ethics Committee (REF 07/Q0603/29) and the trial was conducted according to the Declaration of Helsinki. In this prospective cohort observational study, blood samples were collected at admission and over the first 3 days after injury, and clinical characteristics, injury severity, mechanism of injury, transfusion of blood products and outcome at 28 days post-injury were obtained from patients’ records.

For the first study presented here (discovery), we included 8 patients with isolated TBI, head abbreviated injury score (AIS)4 (severe injury) and other AIS0. For the validation samples, we selected 5 TBI patients with head AIS3 (serious injury) and other AIS0. For both groups, controls with head and other AIS0 were selected, and as much as possible age and sex matched to TBI patients – within the limitations of the available biobank samples. For the first study the average age was very comparable between TBI and control patients, whereas in the second study the TBI patients had an older average age, but the difference between them and controls was not statistically significant.

### Blood samples

Blood samples were collected at admission (0 h), 24 ± 2 h and 72 ± 12 h post admission, from either a central, arterial or large bore peripheral line, where available, or the femoral vein or antecubital fossa. Blood was collected in citrate tubes, and centrifuged (1750 g, 10 min). Plasma was centrifuged again under the same conditions. The plasma and cell pellet were stored at −80°C until analysis. Since not all samples were available for every patient, supplemental Table S1 shows an overview of the sample availability.

### Lipidomics

LC-MS Lipidomics was performed according to an established protocol.^31^ In brief, 50 μL of plasma was thawed at 4°C for 2 hours before dilution with H_2_O (1:1 v/v) in a 96-well plate. Protein precipitation was performed by the addition of 4 parts of isopropanol including reference standards used for analytical performance monitoring (supplementary table S2). After mixing (1400 rpm, 2 h, 4°C), samples were centrifuged (3486 g, 10 min, 4°C) and supernatant transferred to a fresh 96-well plate for injection (2 μL) onto the LC-MS system.

Lipidomic profiling was performed using reversed-phase chromatography on a Waters Acquity LC system and a Waters BEH C8, 2.1 × 100 mm, chromatographic column (both Waters Corp, Manchester, UK). Chromatographic separation was performed at 55°C using a linear gradient (supplementary table S3) consisting of phase A; 50:25:25 mixture of water:acetonitrile:isopropanol with 5 mm ammonium acetate, 0.05% acetic acid, and 20 µM phosphoric acid and phase B; 50:50 acetonitrile:isopropanol with 5 mM ammonium acetate, 0.05% acetic acid. The LC was coupled to a high resolution Xevo G2‐S TOF mass spectrometer (Waters Corp). Analysis was performed in both positive and negative ion modes. MS settings were as follows: Capillary voltage = 2.0 kV (Pos), 1.5 kV (neg); sampling cone = 25 V; source temperature = 120°C; desolvation temperature = 600°C; desolvation gas flow = 1000 L/min; cone gas flow = 150 L/min, mass range = 50–2000 Daltons.

Replicate extractions of pooled biological sample underwent analysis at consistent intervals throughout the analytical run to act as quality control samples and to monitor analytical performance and precision throughout the data acquisition. Full analytical instrument details are presented in supplemental information and supplemental Table S3.

### Lipidomics data pre-processing

Raw data was converted to the mzML open-source format using the MSConvert tool in ProteoWizard.^32^ Metabolite signal extraction was performed using PeakPantheR,^33^ an open-source R package to detect, integrate and report pre-defined annotated lipids. Lipid annotations for implementation in PeakPantheR have been described by Lewis et al.^31^ To summerise, annotations were achieved through the manual inspection of MS/MS fragmentation patterns and rules-based lipid fragmentation to achieve identification of lipid headgroup and sidechain combinations. This included the matching of accurate mass MS/MS fragmentation measurements to reference spectra from online databases (LIPID MAPS,^34^ Metlin^35^ and Human Metabolome Database; HMDB^36^) and where chemical reference materials were commercially available, direct matching of chromatographic and spectral signatures between reference standards and biological samples.

Elimination of potential run-order effects and filtering of the extracted metabolites was performed using the nPYc-Toolbox, an open-source package for data pre-processing.^37^ Lipid features were removed that had relative coefficient of variance in quality control (QC) samples greater than 20% and demonstrated linearity (>0.8) in a dilution series of QC. Annotated lipid features from positive and negative ionisation modes were concatenated into a single matrix for each batch. Annotated features were only kept for the final dataset if they were matched across each dataset. This consisted of 387 lipid feature variables in 31 discovery samples and 36 validation samples. Peak area integrals underwent median factor normalisation to standardise the peak integrals across both batches prior to data analysis.

### Lipidomics data analysis

Lipidomics data analysis was performed in R (v4.2.2) and R Studio (v2023.03.0). Lipidomics data from the discovery and validation batches were treated independently throughout the data analysis phase of the study. To evaluate precision of data acquisition, unsupervised principal component analyses (PCA) were performed using the rOPLS package.^38^ Supervised partial least squares analyses (PLS) that considered all four groups and pairwise orthogonal projections to latent structures discriminant analyses (OPLS-DA) (Control_0 to TBI_0; Control_0 to TBI_24; Control_0 to TBI_72) were also performed using the rOPLS package for classification between groups.

Univariate *t* tests were performed using the ggpubr package in R (Control_0 to TBI_0, Control_0 to TBI_24 and Control_0 to TBI_72 groups). To visualise the univariate output, volcano plots were generated in R using in-house scripts, displaying log2 fold change (x) and log2 -p values (*t* test) (y). Due to the discovery nature of the study, and high ratio of lipid variables to samples in the dataset, corrections for multiple testing were not performed.

### Omega-3 index

Fatty acids were extracted from 250 µl cell pellet using the method described by Bell and colleagues,^39^ based on a modified Bligh and Dyer extraction, using methanol and chloroform. The resulting oily residue was transesterified using BF_3_-methanol.^40^ Fatty acid methyl esters were analysed by gas chromatography with flame ionisation detector (7820 A; Agilent Technologies, Santa Clara, CA, USA), using an Omegawax^TM^ column (30 m × 0.25 mm, d_f_ 0.25 µm, Supelco). In total, 21 fatty acids were identified, compared to analytical standards and quantified, with corrections applied for variations in the detector response. The O3I was calculated as the sum of EPA and DHA expressed as percentage of total fatty acids.^41^

### Neurofilament light

NFL in plasma was quantified using a singleplex sandwich immunoassay (Human NFL R-PLEX, MSD), according to manufacturer’s instructions. Briefly, the plate was coated with biotinylated capture antibody for 1 h, before incubation with samples or standards for 1 h. After washing, SULFO-TAG detection antibody was incubated for 1 h, followed by addition of read buffer and electro-chemiluminescent detection on a MESO QuickPlex SQ120 instrument. Samples were analysed using Discovery Workbench V4, with lower limit of detection determined as 5.73 pg/ml. Samples were analysed in duplicates. One control sample in the discovery set had to be excluded based on sample availability, therefore n = 7.

### Cytokines

Plasma cytokines (IFN-γ, TNF-α, IL-1β, IL-2, IL-4, IL-6, IL-8, IL-10, IL-12p70, IL-13) were quantified using a validated sandwich immuno-multiplex assay (V-PLEX Proinflammatory Panel 1, MSD), according to manufacturer’s instructions. Briefly, samples and standards were added to the multiplex plate containing capture antibodies and incubated for 2 h. After washing, SULFO-TAG detection antibody was incubated for 2 h, followed by addition of read buffer and electro-chemiluminescent detection on a MESO QuickPlex SQ120 instrument. Samples were analysed using Discovery Workbench V4. Lower limits of detection were determined for each of the ten cytokines (supplemental Table S4). Samples were analysed in duplicates and run at the same time. Two control samples from the discovery set (n = 5), one control sample from the validation set (n = 4), and one TBI AIS4, 72 h samples (n = 7) had to be excluded due to sample availability.

### Statistical analysis

Guidelines for strengthening the reporting of observational studies (STROBE) were followed.^42^ N-number is provided in cases where data sets are not complete due to sample availability. Statistical analysis was performed using GraphPad Prism (Version 9.5.1). Data was tested for normality using the D’Agostino and Pearson test. Groups were compared using *t* test/Mann-Whitney U test or one-way ANOVA/Kruskal-Wallis test, with Tukey’s or Dunn’s post-hoc test, respectively. Outliers were detected using Grubb’s test. Spearman correlation was used for correlations. Data are presented as means ± SD or median [IQR] and considered statistically significant at p < 0.05.

## Results

### Participant characteristics

Participants’ characteristics between the discovery and validation sets are similar, besides the difference in Head AIS (4 for the discovery set and 3 for the validation set of samples). On average patients were middle aged (41.0 ± 19.8 years vs 33.4 ± 13.1 years), and predominantly male. TBI patients had lower GCS scores than controls, stayed longer in hospital and 30% of patients died. Further participants’ characteristics are detailed in Table 1.

**Table 1.** Participant characteristics.

	Discovery set		Validation set	
Injury type	TBI	Control	TBI	Control
N=	8	8	5	5
Age	41.0 ± 19.8	44.6 ± 21.2	54.6 ± 21.7	33.4 ± 13.1
Male/Female	7/1	6/2	3/2	3/2
Head AIS	4 [0]	0 [0]	3 [0]	0 [0]
Other AIS	0	0	0	0
ISS	16 [0]	0 [0]	9 [0]	0 [0]
GCS at admission	11 [11]	15 [3]	10 [9]	15 [0]
-mild (13–15)	3	6	1	5
-moderate (9–12)	2	1	2	0
-severe (3–8)	3	1	2	0
Days till discharge or death	8.8 ± 7.1	1.1 ± 1.2	13.2 ± 10.4	0.8 ± 0.7
Outcome at 28 days (dead/alive)	2/6	0/8	2/3	0/5
Mode of injury				
- Fall	3	0	0	0
- RTC	1	0	1	4
- HtH	0	6	1	1
- GSW	0	2	2	0
- Assault	4	0	1	0
Blunt/penetrating	8/0	8/0	4/1	5/0
Transfusions				
-Pre-baseline				
Crystalloids	3	0	1	0
Hypertonic saline	0	0	1	0
-0–12 h				
Crystalloids	8	2	2	1
Hypertonic saline	0	0	1	0
Platelets	0	0	1	0
PRBCs	1	1	0	0
-12–24 h				
Crystalloids	6	0	2	2
PRBCs	1	0	1	0

AIS: abbreviated injury scale; GCS: Glasgow Coma Scale; GSW: gunshot wound; HtH: hit to head; ISS: Injury Severity Score; PRBCs: packed red blood cells; RTC: road traffic collision.

### Lipidomics

**Figure 1.**
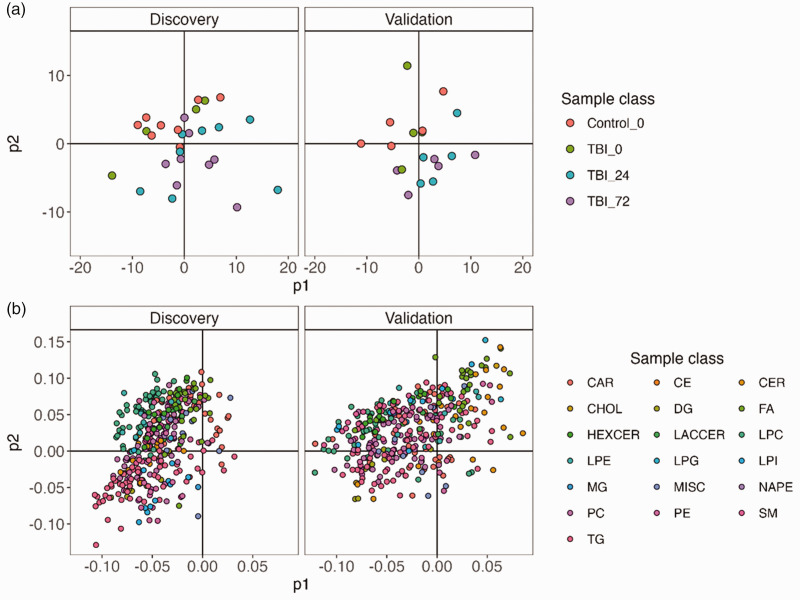
Principal component analysis. (a) PCA scores plots of each cohort, coloured by sample class. The plot indicates a signature of TBI, with each class clustering on the plot. TBI 24 h, and TBI 72 h cluster away from Control indicating a multivariate signature of injury (b) corresponding PCA loading plots for each cohort, coloured by lipid class. The plot indicates the PCA scores signature is driven by specific clusters of lipid classes. CAR: acylcarnitine; CE: cholesterol ester; CER: ceramides; CHOL: cholesterol; DG: diacylglycerol; FA: fatty acid; HEXCER: hexosylceramide; LACCER: lactosylceramide; LPC: lysophosphatidylcholin; LPE: lysophosphatidylethanolamine; LPG: lysophosphatidylglycerol; LPI: lysophoasphatidylinositol; MG: monoacylglycerol; MISC: miscellaneous; NAPE: n-acylphosphatidylethanolamine; PC: phosphatidylcholine; PE: phosphatidylethanolamine; SM: sphingomyelin; TG: triglyceride.

The dataset consisted of 387 lipid variables matched across the discovery and validation batches. Data quality was assessed by evaluating the distribution of replicate analyses of a pooled quality control in PCA. The PCA scores plot revealed pooled quality control clustering compared with study samples, indicating reproducible data acquisition for both the discovery and validation studies (supplemental Figure S1). When colouring the PCA scores plot by sample class distinct groups were present in both cohorts, with the control group clustering with TBI 0 h, and the TBI 24 h clustering with the TBI 72 h group indicating multivariate similarity in lipidomic profiles (Figure 1(a)). Evaluation of the corresponding loading plot revealed LPC, PC and HexCer species in the upper left quadrant, indicating their influence on the scores separation (Figure 1(b)).

**Figure 2.**
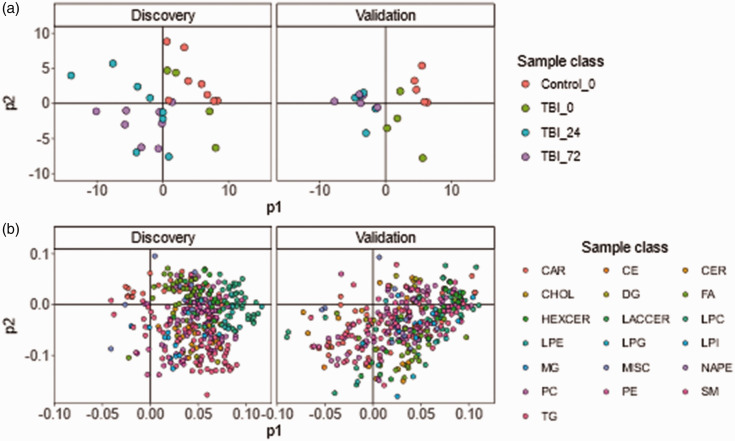
PLS analysis. (a) PLS scores plot of each cohort, indicating clustering by sample class with a trend of time from injury more distinct from Control and (b) corresponding PLS loadings plots for each cohort, coloured by lipid class. The plot indicates the PLS scores signature is driven by specific clusters of lipid classes, specifically LPC, PC and HexCer species as main drivers for separation. Loadings data for each lipid can be found in supplemental Table S5. CAR: acylcarnitine; CE: cholesterol ester; CER: ceramides; CHOL: cholesterol; DG: diacylglycerol; FA: fatty acid; HEXCER: hexosylceramide; LACCER: lactosylceramide; LPC: lysophosphatidylcholin; LPE: lysophosphatidylethanolamine; LPG: lysophosphatidylglycerol; LPI: lysophoasphatidylinositol; MG: monoacylglycerol; MISC: miscellaneous; NAPE: n-acylphosphatidylethanolamine; PC: phosphatidylcholine; PE: phosphatidylethanolamine; SM: sphingomyelin; TG: triglyceride.

This finding was compounded using supervised PLS which enhanced the group clustering (Figure 2(a)) and indicated an order of increasing variation from control < TBI 0 h < TBI 24 h and TBI 72 h. Supporting loadings plot indicated LPC and PC and HexCer species as the main drivers of the scores distribution (Figure 2(b), supplemental Table S5). Clustering of TBI 24 h and TBI 72 h in the PLS analyses indicated a similar multivariate lipidomic signature between both timepoints that was consistent in both discovery and validation datasets.

Pairwise orthogonal projections to latent structures discriminant analyses (OPLS-DA) analyses (Control vs TBI 0 h; Control vs TBI 24 h; Control vs TBI 72 h) indicated successful multivariate classification that differentiated the TBI group from control at each timepoint (supplemental Figure S2). Loadings data for each lipid in the model is also presented in supplemental Table S6 and includes specific details of lipid species and their component sidechains.

**Figure 3.**
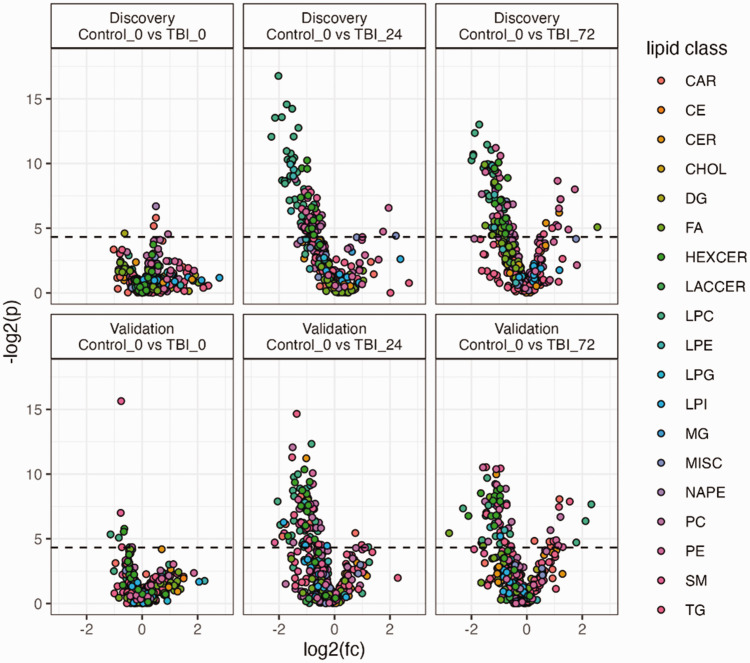
Volcano plots. Volcano plot presenting log2(fold change) vs log2(t test p). Each point represents a lipid variable coloured by class. Data is presented for the discovery and validation TBI samples. Class independent plots for LPC, PC and HexCer are presented in supplemental Figures S3–S5. CAR: acylcarnitine; CE: cholesterol ester; CER: ceramides; CHOL: cholesterol; DG: diacylglycerol; FA: fatty acid; HEXCER: hexosylceramide; LACCER: lactosylceramide; LPC: lysophosphatidylcholin; LPE: lysophosphatidylethanolamine; LPG: lysophosphatidylglycerol; LPI: lysophoasphatidylinositol; MG: monoacylglycerol; MISC: miscellaneous; NAPE: n-acylphosphatidylethanolamine; PC: phosphatidylcholine; PE: phosphatidylethanolamine; SM: sphingomyelin; TG: triglyceride.

Results of univariate *t* test analysis for both discovery and validation datasets are presented in supplemental Table S7. The results are visualised in volcano plots in Figure 3 that display log2 fold change (x) vs -log2 *p* (y) and for each of the lipid variables with point colour defined by lipid class. Class specific volcanoes plots for LPC, PC and HexCer are presented in supplemental Figures S3–5. The univariate analyses revealed few significant lipids at the 0 h timepoint, but a distinct pattern of significantly lower level of LPC, HexCer and PC at the 24 h and 72 h timepoints in both discovery and validation cohorts. Individual standard error plots for exemplar lipids from LPC, PC and HexCer classes are presented in supplemental Figure S6.

**Figure 4.**
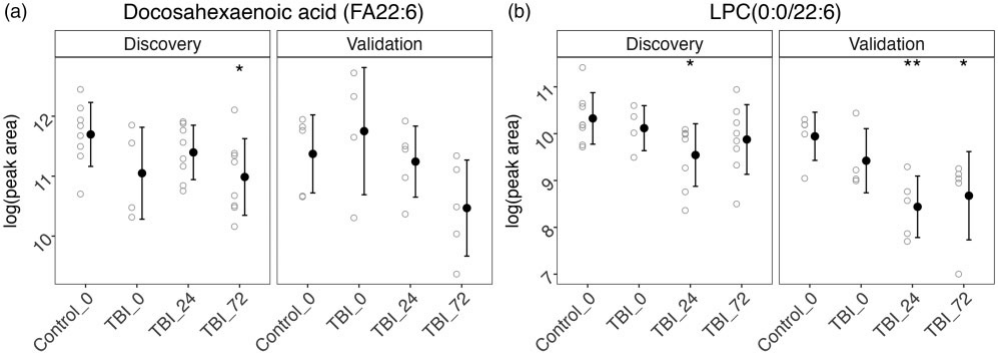
DHA containing species. Standard error plots for (a) DHA and (b) LPC (0:0/22:6).

Univariate *t* test results for plasma DHA revealed significantly lower levels at TBI 72 h compared to controls in AIS4 patients (discovery; Figure 4(a)). Similarly, AIS3 patients had lower, but not statistically significant levels of plasma DHA at 72 h. LPC with DHA sidechain (LPC (0.0/22:6)) data also showed lower levels at 24 and 72 h post injury, compared to controls (Figure 4(b)). These differences were significant at 24 h in AIS3 and AIS4 and at 72 h in AIS3 patients. Plots for other omega-3 and omega-6 fatty acid species showing similar patterns can be found in supplemental Figure S7.

### Omega-3 index

**Figure 5.**
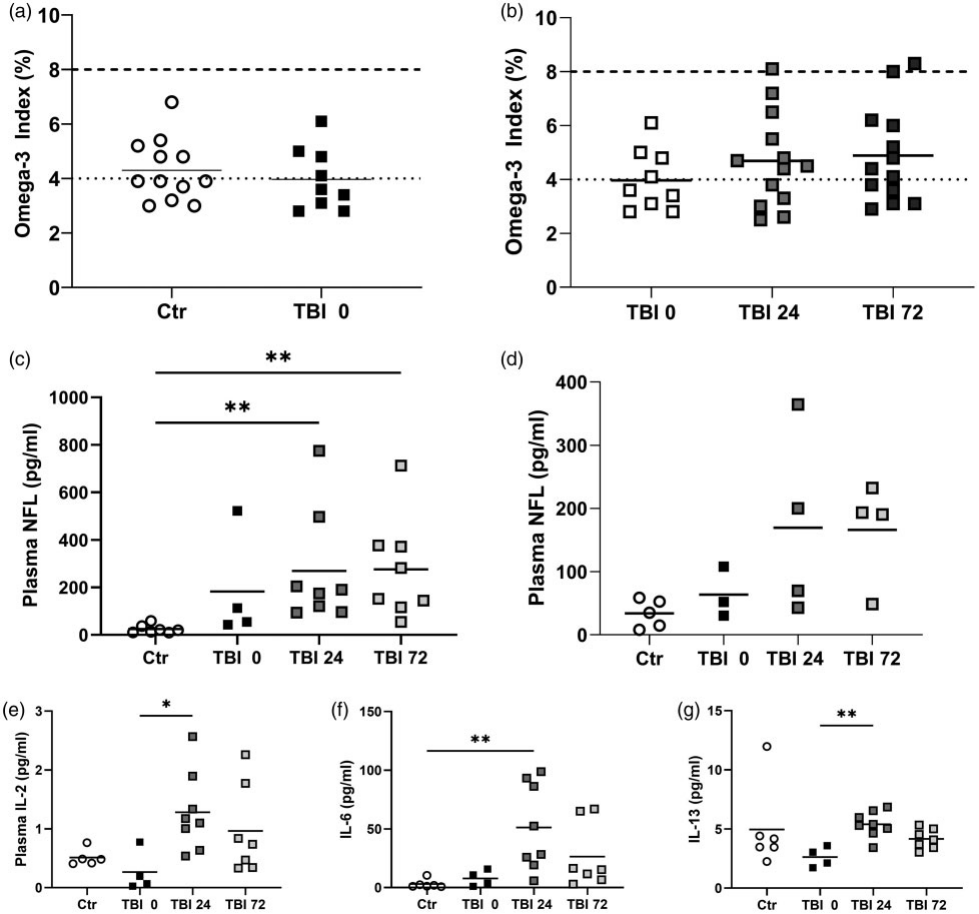
Biomarker analyses. There was no significant difference between (a) the baseline O3I of control, TBI AIS3 and AIS4 (p = 0.5100), and (b) over the 3 days in TBI AIS3 and AIS4 patients combined (p = 0.4220). Plasma NFL levels in (c) TBI AIS4; and (d) TBI AIS3. Compared to controls, NFL levels were significantly higher at 24 h and 72 h in TBI AIS4 patients. **p < 0.01. Plasma cytokine levels after TBI: (e) TBI AIS4 plasma IL-2 levels; (f) TBI AIS4 plasma IL-6 levels and (g) TBI AIS4 IL-13 levels. *p < 0.05; **p < 0.01.

The baseline O3I of patients did not differ significantly between groups and was notably low (control: 4.3 ± 1.1 vs TBI AIS3 and 4: 4.0 ± 1.1; p = 0.5100; Figure 5(a)). There was no significant change in O3I over the first three days after injury in TBI AIS4 (p = 0.5180), and TBI AIS3 (p = 0.4664) (data not shown). Figure 5(b) shows the lack of significant change in O3I over time in all TBI patients. The red blood cell DHA level as percentage of total fatty acids did not change over the time-course in TBI AIS3 or AIS4 patients. Baseline red blood cell DHA levels were control: 3.6 ± 0.9 vs TBI AIS3: 3.4 ± 0.9 vs TBI AIS4: 2.9 ± 1.1, and did not differ significantly (p = 0.4928).

### NFL

There was a significant difference in NFL levels (p = 0.0017), with TBI AIS4 patients having much higher levels at 24 h (269.0 ± 241.9 pg/ml; p = 0.0046) and 72 h (276.1 ± 212.9 pg/ml; p = 0.0037) compared to controls (24.18 ± 17.85 pg/ml; n = 7, due to sample availability; Figure 5(c)). For TBI AIS3, one patient had abnormally high NFL levels, which were excluded after testing for outliers. In this group, an increase in NFL can be seen from 63.61 ± 39.93 pg/ml at 0 h to 169.5 ± 147.0 and 166.3 ± 80.69 pg/ml at 24 and 72 h, respectively (Figure 5(d)). However, this increase was not statistically significant. NFL levels at 24 h in AIS3 and AIS4 patients as well as in control patients were significantly correlated with the GCS of the patient (r = −0.4537; p = 0.03).

### Cytokines

Ten different cytokines were measured (supplemental Table S8). For IL-6, there was a significant difference in the discovery set (p = 0.0021), with TBI AIS4 (n = 7) patients having significantly higher levels at 24 h than controls (p = 0.0015; n = 5; Figure 5(f)). IL-6 levels were also significantly correlated to GCS of all patients (r = 0.05420, p = 0.008). IL-2 and IL-13 plasma concentration also increased significantly (p = 0.0191 and p = 0.00094, respectively) from 0 h to 24 h in AIS4 patients (Figure 5(e) and (g)). No significant differences were noted for any cytokine for AIS3 patients.

### Lipid correlations

**Figure 6.**
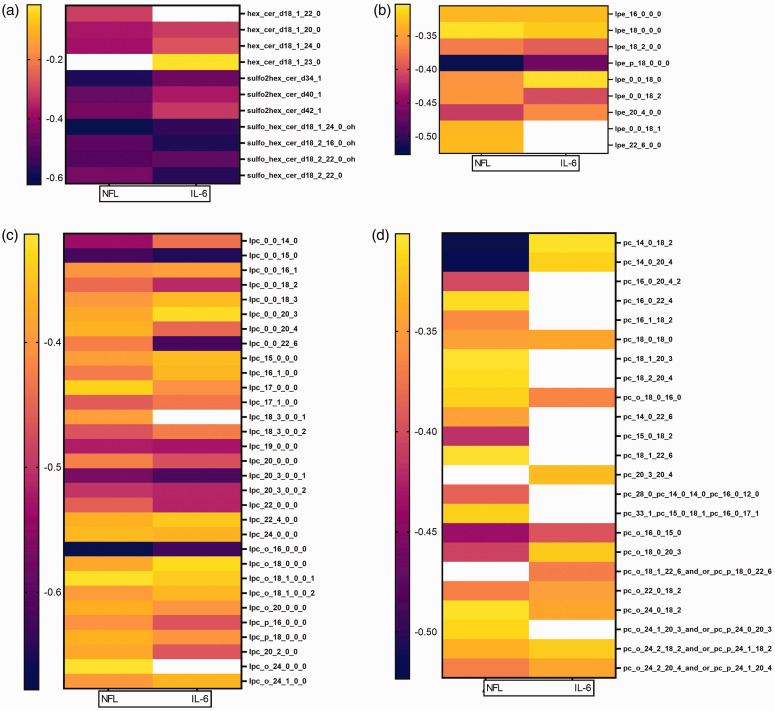
Heatmap of lipid correlations with NFL and IL-6. Strength of significant correlation of (a) HexCer, (b) LPE, (c) LPC, and (d) PC with NFL and IL-6. White squares indicate no significant correlation.

Inflammation and neuronal damage are hallmarks of TBI. Therefore, identified lipid classes, which separated TBI patients from controls, were correlated with these markers. Ten of the identified HexCers (20), 31 of the identified LPCs (48), 9 of the identified LPEs (11), and 21 of the identified PCs (66) were significantly correlated with NFL levels. Similarly, 8 of the identified HexCers (20), 29 of the identified LPCs (48), 7 of the identified LPEs (11), and 12 of the identified PCs (66) showed significant negative correlations with IL-6. The correlation strength of significant correlations can be seen in Figure 6. Supplemental Figure S8 and S9 show the top 6 correlations of lipids with NFL and IL-6, respectively. LPC 0:0/22:6 had strong negative correlations with IL-6 and NFL levels (r = −0.6477, p < 0.001; r = −0.426 p = 0.004; respectively). Similarly, lower plasma DHA FFA levels were correlated with higher levels of NFL (r = −0.3083, p = 0.04), while plasma DHA FFA was not significantly correlated with IL-6 (r = −0.2024, p = 0.19).

## Discussion

In the present study we identify a lipid signature of the acute phase of human TBI, characterised by changes in PL, LysoPL and HexCer species, and show the correlation of this lipid signature with markers of neuronal injury and neuroinflammation. Furthermore, we show a decrease in plasma DHA and specifically in species containing this acyl residue, e.g. LPC (0:0/22:6), which are critically involved in the brain uptake of this fatty acid. We also show that all patients presented at admission a low O3I, which remained low during hospital stay.

Lipids account for more than 50% of human brain dry weight. Cellular membranes are comprised mainly of proteins and lipids, and PL, along with cholesterol and sphingolipids, are major lipid components. PL can be produced by different biosynthetic routes, and a key pathway utilizes LysoPL and acyl-coenzyme A as substrates. There are PL with various head groups: PC, phosphatidylethanolamine (PE), phosphatidylserine (PS), phosphatidylinositol (PI), phosphatidylglycerol (PG). An analysis of mouse CNS lipidome shows that PC and PE are major species present in neurons, whereas astrocytes are enriched in PS and PI, and microglia in sphingomyelin and PG; HexCer, which are structural components of myelin, are enriched in mature oligodendrocytes.^43^ The authors also show that LysoPL species, such as LPC, increase in concentration as a function of neuronal differentiation. LPC species are amphiphilic PL, derived from PC. Unsaturated LPC is bound to albumin in the circulation and the acyl group can move from the sn-2 to the sn-1 position at physiological pH. Nguyen and colleagues^44^ identified the protein which acts as a major transporter for DHA into the brain, i.e. the major superfacilitator family Mfsd2a transporter, and showed that it facilitates the import of DHA into the brain using preferentially the fatty acid in an LPC form. Therefore, plasma species such as LPC (0:0/22:6) have a major role in maintaining an adequate supply to the CNS of this fatty acid, which is essential for neurodevelopment and normal brain function. Synaptic PL are highly enriched in DHA.^45^

Quehenberger et al.^46^ carried out a comprehensive lipidomic analysis of healthy human plasma and showed that the main PL species are PC and PE. In our study we show a decline in PC levels after TBI, as well as in LPC and HexCer species. A key question is whether changes seen acutely after TBI directly reflect changes in the central compartment and/or compensatory changes in lipid pools from the periphery. i.e an attempt to mitigate the impact of injury.

The pathophysiology of TBI reflects processes that occur during the primary injury phase and then the secondary injury cascade. In the former, the injury leads to major disruption of the tissue, with loss of neuronal and non-neuronal cells and major vascular disruption, haemorrhage and ischaemia, and major blood-brain barrier disruption. The secondary injury cascade amplifies the consequences of the primary event, and includes elevated glutamate release, leading to excitotoxicity, oxidative stress, compromise of cerebral energy metabolism, and neuroinflammation – involving both local microglia and infiltrating peripheral cells, such as macrophages.^47–49^ Within the first hour after TBI, there is significant activation of phospholipases, as shown in experimental TBI models in mice and rats.^17,50,51^ The PL cleavage by phospholipases releases a variety of FFA and also DAG, and the intensity of this process is reflected in clinical outcome in human TBI; thus, CSF FFA levels measured within 2 days after injury were inversely correlated with the Glasgow Outcome Score at hospital discharge.^18^ Human CSF contains (in order of quantitative enrichment) the following species: PC, SM, PE, LPC, PI and PS.^52,53^ Pasvogel et al.^54^ assessed the CSF dynamics of these species after severe TBI and showed an increase in all species after injury, with the highest LPC level already on day 1 and the highest PC level at 4 days. These processes are likely to reflect the global demise of cellular membranes- early necrotic and then apoptotic cell death- and the ongoing changes in grey and white matter during the secondary injury phase, still detectable 96 h after injury. Therefore, overall, after injury there is a first wave of loss of PL as structural components of cells that are destroyed by the direct impact of the injury, and also an amplification of the enzymatic degradation of the PL, with formation of FFA. The loss of LPC from the brain parenchyma would ultimately compromise the availability of long-chain fatty acids such as DHA, as local brain synthesis of these fatty acids is very limited.^55–57^ Furthermore, the metabolism of the released long-chain fatty acids, such as the omega-6 arachidonic acid (AA; 20:4n-6) and the omega-3 EPA and DHA, will give rise to complex changes in lipid mediators derived from these fatty acids, with greater pro-inflammatory, or more anti-inflammatory and pro-resolving properties, respectively.^9,20,58^ It could be speculated that these changes in the lipids in the central compartment could attract a compensatory influx of lipids from the peripheral compartment, thus leading to the decreases we observe in plasma LPC and PC over the first 3 days. Concerning HexCer, there is limited information on the plasma or CSF dynamics of this species. It has been shown to increase in the CSF during disease progression in multiple sclerosis, indicating white matter damage.^59,60^ The movement of lipid species such as HexCer, and also PC or LPC from the brain/CSF compartment to plasma, may be a consequence of the blood-brain barrier disruption and its leakage post-injury, and also to some extent involve the glymphatic system.^61^ In contrast, the transfer of such species from plasma to the brain is a much less characterised process.

We show that the levels of NFL are elevated in TBI patients (in particular at AIS4) and remain high at 3 days post-injury, supporting previous reports on the validity of this marker as an index of neuronal injury.^7,62^ This first study was focused on NFL as a marker of neuronal demise, but in future studies, other biomarkers of TBI, such as glial fibrillary acidic protein (GFAP) and ubiquitin C-terminal hydrolase (UCH-L1) or S-100 beta(β), would be very interesting to monitor, to give a more comprehensive indication of neuronal and non-neuronal damage. Furthermore, the early injury phase in our TBI patients was also characterized by higher plasma levels of cytokines such as IL-6, IL-2 and IL-13 compared to controls, reflecting an inflammatory response to TBI which is linked to the local activation of microglia and astrocytes in the brain parenchyma and also to the peripheral inflammatory responses.^24,63^ Kossmann et al.^64^ reported a prolonged increase in IL-6 in CSF and serum in severe TBI, with the peak serum levels being correlated with high levels of acute-phase proteins. In a recent review of this cytokine in TBI, Ooi and colleagues^65^ propose that the early increase in IL-6 levels after injury is correlated with poor outcome. In our study, we also report an increase in plasma IL-2, a cytokine with pleiotropic effects and a major regulator of Treg cells.^66^ The sequence of cellular infiltration in experimental TBI encompasses early neutrophil influx followed by monocytes/macrophages and a later arrival of T- and B-cells.^67,68^ Yshii and colleagues^69^ report that the administration of IL-2 after TBI has neuroprotective effects. We also report an increase in plasma IL-13, a cytokine expressed at synapses^70^ and involved in cognitive function.^71^ It has been shown that this cytokine is upregulated after TBI in human brain tissue and in the CSF, within the first 24 h post-injury, and a similar increase was also seen in mouse TBI, in the cytokine and its receptor IL-13 Ra1, within 3 h after the injury; furthermore, the application of IL-13 protects against excitotoxicity *in vitro*, therefore, this early increase in the cytokine and its receptor post-injury could be hypothesized to have a neuroprotective impact. The repeated intranasal administration of IL-13 after TBI in mice, leads to neurological improvement and reduction of the inflammatory response, and also reduced white matter injury.^72^ Similarly, the post-stroke treatment with IL-13 in mice has been shown to improve neurological recovery and reduce tissue loss, in parallel with an increased polarization of microglia and infiltrating macrophages towards an anti-inflammatory profile.^73^ It will be interesting in future studies to assess a much more comprehensive panel of cytokines, to gain more insight into the dynamics of the peripheral inflammatory markers. It is also important to note studies that report correlations between lipids and cytokines in brain tissue. Pulliam et al. have studied in a rat model of mTBI – a closed head impact which induces a diffuse injury, correlations in brain tissue 24 h post-injury between the lipidomic profile and a panel of cytokines.^27^ They also compared single and repeated injury (3 impacts), and potential differences between males and females. Most of the FFA, DAG, and SM decreased in cortical tissue in both the single and repetitive mTBI animals. There was a more significant decrease in lipids in the repeated injury group, that may be a result of the higher level of injury. They report that 78% of the FFA and 73% of the SM decreased in the repeated injury group relative to the sham control group. In the repeated injury group, they also report a significant decrease, relative to the sham controls, in the cytokines IL-1β, IP-10, TNF-α, and RANTES. The results showed negative correlations between lipid changes and eotaxin, TNF-α, IP-10, and RANTES. All these observations highlight the complexity of the interactions between lipids and cytokines in different injuries and different tissues. In a review focused on stroke, Adhibatla et al. indicate possible mechanistic links between changes in lipids and inflammatory markers after acquired brain injury: for example, a key enzyme involved in PC synthesis is phosphocholine cytidyltransferase, and TNF-α inhibits this enzyme, with potential deleterious impact on the synthesis of PC.^74^ TNF-α could also stimulate phosphorylation of cytosolic phospholipase A_2_ and through activation of the enzyme initiate the AA cascade, enhancing the pro-inflammatory signal in tissue. IL-1 and TNF-α could also inhibit SM synthesis through activation of sphingomyelinases.

It is notable that all patients included in this study, whether controls or TBI patients, had a rather low O3I (average value <4.5%, compared to the optimal 8% and above), which remained low over the 3 days post-admission. The O3I is directly determined by dietary intake and is also influenced by inheritable characteristics.^75^ It is inversely related to sudden death, depression, acute coronary syndromes and total mortality, and these beneficial effects are likely to be directly linked to specific effects of EPA and DHA on endothelial function, inflammation, platelet function and heart rate. A study in US veterans has shown that after a TBI the risk of cardiovascular disease is increased, with the risk being highest shortly after injury, but remaining high for years after TBI.^76^ Furthermore, the O3I of red blood cells is correlated, amongst others, with brain DHA levels,^77^ indicating that patients in this trial also had low brain omega-3 fatty acid levels. This might be detrimental for TBI patients and their recovery. Experimental studies have shown significantly slower recovery from post-TBI motor deficits and cognitive deficits, as well as higher levels of anxiety in DHA-depleted mice,^78^ while it has been shown that pre- and post-injury treatment with omega-3 fatty acids is protective against neurotrauma.^79^ Therefore, this allows us to suggest that by measuring the O3I at admission, should the index be found to be low, supplementation with omega-3 fatty acids during hospital stay could be carried out and may have long-term beneficial effects.

This was an exploratory study, in which plasma and red blood cell samples from TBI patients with various severity, from a trauma biobank, were analysed and compared to controls. Control patients were assessed and had a head AIS of 0 and no other injuries. Nevertheless, these participants had reasons to be present in hospital, which should be considered. Due to the biobank origin of the samples, we cannot provide further information about the severity of the injury or the outcome of the patients besides the initial classification, measured NFL levels and the dead or alive outcome at one month. In future studies, it would be interesting to establish whether the identified lipid signature also correlates with, and could therefore predict, clinical/functional outcome measurements later, including for example the Extended Glasgow Outcome Score or the Functional Status Examination.^80^ We appreciate the small sample size of our study, which was a pragmatic approach for a pilot study.^81^ Nevertheless, it should be highlighted that results found in TBI AIS4 patients were validated in a second independent analysis of a TBI patient group with a less severe injury (AIS3). While the results measured over the first three days of injury clearly show a lipid signature that is present in the acute phase, it cannot be ruled out that these changes persist in the chronic phase of TBI. Emmerich and colleagues showed long-lasting changes in plasma lipids in veterans who have sustained a mTBI while being deployed.^82^ A further limitation is that the current study cannot ascertain the origin of the changes in plasma lipids that were identified. Changes might directly relate to the disruption or breakdown of lipid membranes in the CNS following TBI, which can be detected in the bloodstream because of the leaky blood brain barrier.^83^ However, they might also reflect general changes in the periphery following TBI,^84^ including altered hepatic lipid metabolism.^85,86^ Changes in LPC (0:0/22:6) could also reflect an increased, potentially compensatory, transport of DHA into the CNS, since this is the preferred form of this fatty acid for import into the brain.^44^

TBI is a worldwide problem with 1.4 million people affected per year in the UK alone^87^ and currently there are no effective treatments, with trials failing to produce generalisable interventions.^88^ Menon and Maas hypothesise that part of the problem is the use of the GCS to stratify patients. It is a crude tool with many confounders like the use of sedative medication, which also does not address the disease heterogeneity.^89^ Therefore, there is the need to identify new biomarkers for TBI, which could help with stratification of patients in clinical trials. Here we report a lipid signature which correlated with neuronal damage and inflammatory markers, two of the hallmarks of TBI. To our knowledge, this is the first study demonstrating a link of the lipidomics data with additional markers of TBI, in humans. Additionally, this analysis included patients with varied severities of TBI based on the GCS, providing more inclusive results. Whereas, besides the study by Thomas and colleagues,^90^ most groups performing plasma lipidomic analysis after human TBI focused only on mTBI cases.^14,15,82,91^

To summarise, in this exploratory study on our available biobank samples, we show that there is a distinct systemic lipid signature of the acute phase post-TBI, and it is correlated with neuronal injury and inflammatory responses. This study provides a rationale for future prospective studies in a larger patient cohort, which will increase the value of these first observations. The data suggest that injury leads to a relative deficit in certain PL species and also in DHA. The present study investigated the first 3 days, and the trends observed indicate that these specific changes could continue beyond the first 3 days, giving scope to a restorative intervention that could be implemented as soon as the patients are stable clinically. We recently carried out an interventional study in an experimental model of TBI in mice, using a specialised medical nutrient that provides precursors for PL synthesis and we showed that this intervention leads to much improved neurological outcome and also significant tissue protection and reduction of inflammation.^92^ Therefore, the evidence we present here of disruption of PL metabolism post-TBI from the earliest phase following injury, and the proof-of-concept data in animals showing that it is a targetable process, could open up new therapeutic avenues in TBI management.

## Supplemental Material

sj-pdf-1-jcb-10.1177_0271678X241276951 - Supplemental material for A plasma lipid signature in acute human traumatic brain injury: Link with neuronal injury and inflammation markers Supplemental material, sj-pdf-1-jcb-10.1177_0271678X241276951 for A plasma lipid signature in acute human traumatic brain injury: Link with neuronal injury and inflammation markers by Isabell Nessel, Luke Whiley, Simon C Dyall and Adina T Michael-Titus in Journal of Cerebral Blood Flow & Metabolism

sj-xlsx-2-jcb-10.1177_0271678X241276951 - Supplemental material for A plasma lipid signature in acute human traumatic brain injury: Link with neuronal injury and inflammation markers Supplemental material, sj-xlsx-2-jcb-10.1177_0271678X241276951 for A plasma lipid signature in acute human traumatic brain injury: Link with neuronal injury and inflammation markers by Isabell Nessel, Luke Whiley, Simon C Dyall and Adina T Michael-Titus in Journal of Cerebral Blood Flow & Metabolism

sj-xlsx-3-jcb-10.1177_0271678X241276951 - Supplemental material for A plasma lipid signature in acute human traumatic brain injury: Link with neuronal injury and inflammation markers Supplemental material, sj-xlsx-3-jcb-10.1177_0271678X241276951 for A plasma lipid signature in acute human traumatic brain injury: Link with neuronal injury and inflammation markers by Isabell Nessel, Luke Whiley, Simon C Dyall and Adina T Michael-Titus in Journal of Cerebral Blood Flow & Metabolism

sj-xlsx-4-jcb-10.1177_0271678X241276951 - Supplemental material for A plasma lipid signature in acute human traumatic brain injury: Link with neuronal injury and inflammation markers Supplemental material, sj-xlsx-4-jcb-10.1177_0271678X241276951 for A plasma lipid signature in acute human traumatic brain injury: Link with neuronal injury and inflammation markers by Isabell Nessel, Luke Whiley, Simon C Dyall and Adina T Michael-Titus in Journal of Cerebral Blood Flow & Metabolism

## Data Availability

All data presented are available from the corresponding author on reasonable request (for access please contact Isabell Nessel, i.nessel@qmul.ac.uk at Queen Mary University of London).
