# Supplementary material for: A plasma lipid signature in acute human traumatic brain injury: Link with neuronal injury and inflammation markers
Source: J Cereb Blood Flow Metab. 2024 Aug 26;45(3):443–58. doi: 10.1177/0271678X241276951 (PMC11572080; doi:10.1177/0271678X241276951)
Supplement: sj-pdf-1-jcb-10.1177_0271678X241276951 - Supplemental material for A plasma lipid signature in acute human traumatic brain injury: Link with neuronal injury and inflammation markers [file sj-pdf-1-jcb-10.1177_0271678X241276951.pdf]

Supplementary material

A plasma lipid signature in acute human traumatic brain injury: link with neuronal injury and inflammation markers

Isabell Nessel*, Luke Whiley*, Simon C. Dyall, Adina T. Michael-Titus

Supplementary Tables

Supplementary table S1: Blood sample availability

Time points (Plasma/RBCs)	*TBI (AIS4) discovery*	*Control discovery*	*TBI (AIS 3) validation*	*Control validation*
Admission	4/4	8/8	4/5	5/5
24 h	8/8	N/A	5/5	N/A
72 h	8/8	N/A	5/5	N/A

Supplementary table S2: Internal reference standard mixture

	Standard	*Supplier & Part number*
1	LPC9	Avanti, 855276P
2	PC11	Avanti, 850330P
3	C17	Sigma, H3500
4	PG15	Avanti, 840446P
5	PE15	Avanti, 850704P
6	PS17	Avanti, 840028P
7	PA17	Avanti, 830856P
8	Cer17	Avanti, 860517P
9	DG19	Sigma, 68633
10	PC23	Avanti, 850372P
11	TG15	Sigma, T4257
12	TG17	Sigma, T2151

Supplementary table S3: Reverse phase chromatography gradient

Step	*Time (min)*	*Flow (ml/min)*	*% A*
1	Initial	0.6	99.0
2	0.10	0.6	99.0
3	2.00	0.6	70.0

4	11.50	0.6	10.0
5	12.00	1.0	0.1
6	12.5	1.0	0.1
7	12.55	0.9	35.0
8	12.65	0.8	70.0
9	12.75	0.7	99.0
10	12.95	0.6	99.0
11 (sample load)	15.00	0.6	99.0

Supplementary table S4: Detection limits for cytokines

Cytokine	*Lower (pg/ml)*	*Upper (pg/ml)*
IFN-γ	0.614	1450
TNF-α	0.0900	324
IL-1β	0.0249	584
IL-2	0.0947	1390
IL-4	0.0107	208
IL-6	0.141	683
IL-8	0.0356	580
IL-10	0.0233	381
IL-12p70	0.0688	447
IL-13	0.850	481

Supplementary table S5: Loading data for each lipid in the PLS analysis (Figure 2)

See separate excel file.

Supplementary table S6: Predictive loading data for pairwise OPLS-DA analysis

See separate excel file.

Supplementary table S7: Univariate *t* test results

See separate excel file.

Supplementary table S8: Cytokine results

**Cytokine**	**Discovery set**	**Validation set**
IFN-γ	NS	NS
TNF-α	NS	NS
IL-1β	NS	NS
IL-2	$p = 0.0191$<br>24 h > 0 h	NS
IL-4	NS	NS
IL-6	$p = 0.0021$<br>24 h > Ctr	NS
IL-8	NS	NS
IL-10	NS	NS
IL-12p70	NS	NS
IL-13	$p = 0.00094$<br>24 h > 0 h	NS

NS: not significant

Supplementary Figures

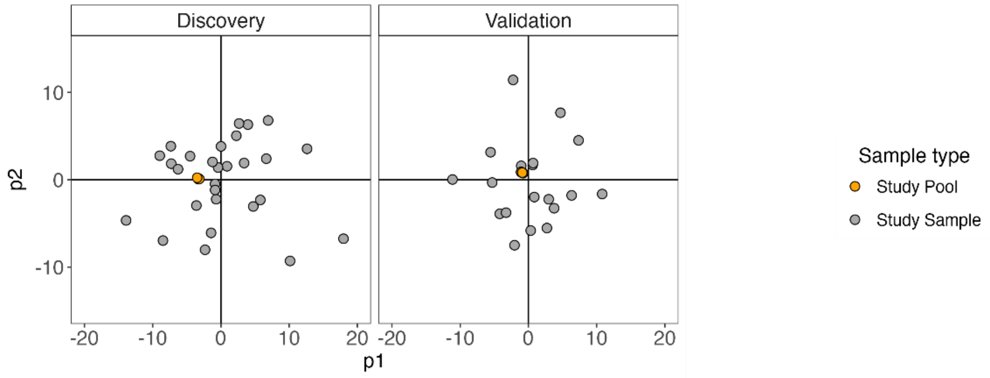

Supplementary figure S1: Quality control scores plot

Scores plots from PCA of the discovery and validation cohorts, pooled quality control samples (orange) demonstrate tight clustering within the study samples (grey) indicating reproducible data acquisition.

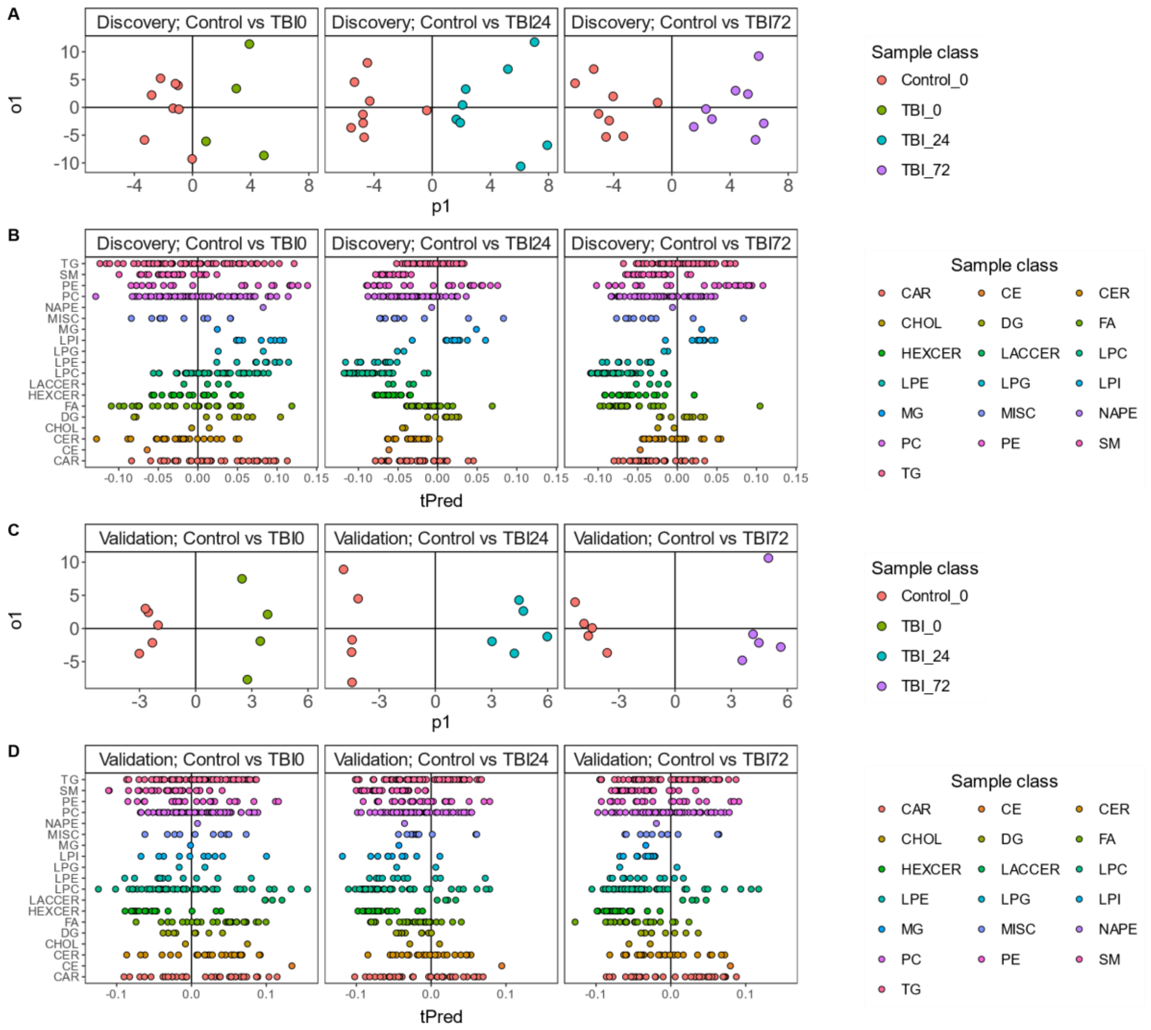

Supplementary figure S2: OPLS-DA analysis

Pairwise OPLS-DA analyses (control vs TBI 0h; Control vs TBI 24h; Control vs TBI 72h). Score plots (A,C) indicate multivariate classification differentiating TBI from control at each timepoint in both discovery and validation. Loadings data (B,D) indicates a shift in lipid signature as time from injury increases, with specific changes in LPC, LPE and HexCer species. Full predictive loading data is presented in supplementary table VI and includes specific details of lipid species and their component sidechains.

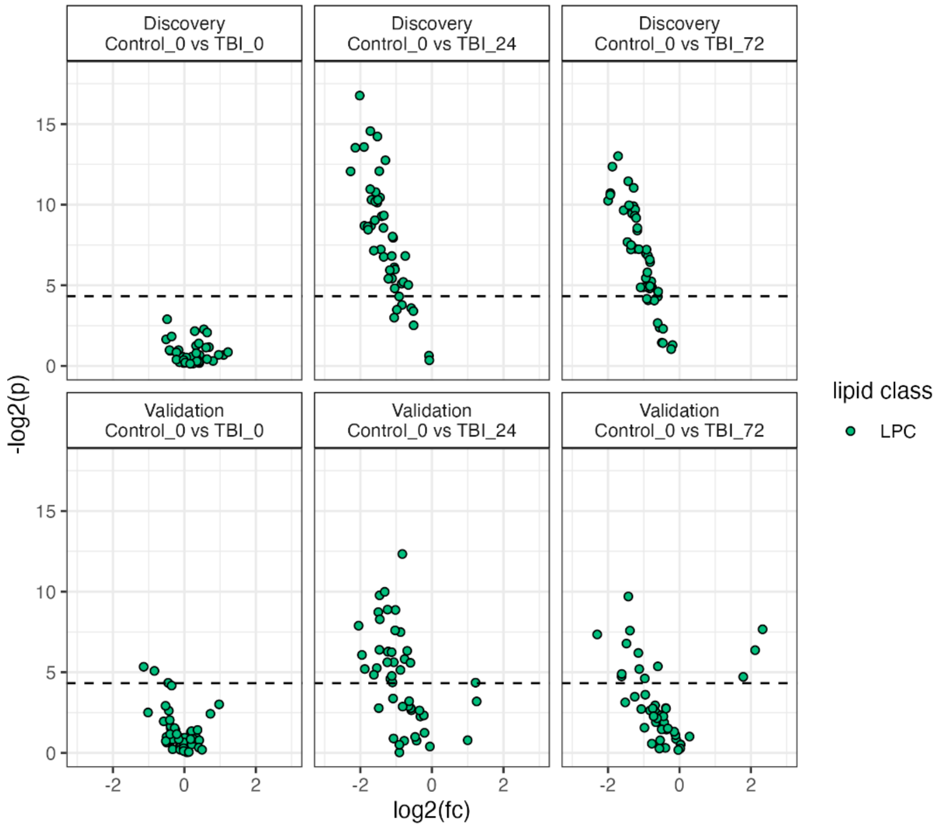

Supplementary figure S3: LPC volcano plots

Volcano plot presenting log2(fold change) vs log2(t test p). Each point represents a LPC variable.

Data is presented for the discovery and validation TBI samples.

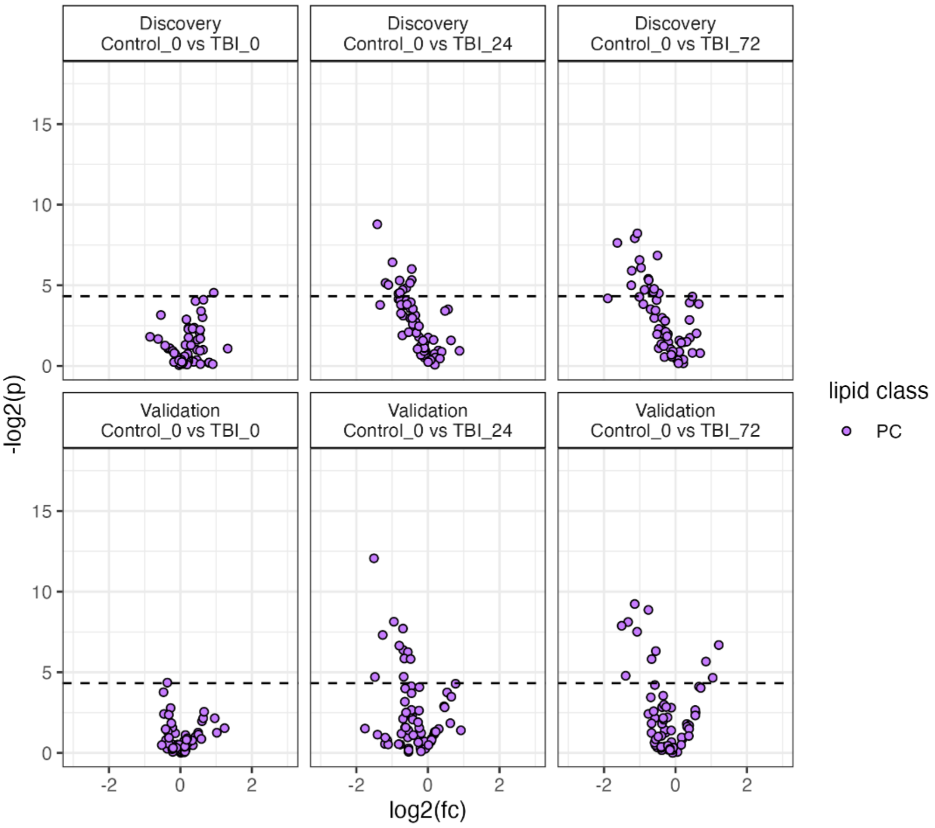

Supplementary figure S4: PC volcano plots

Volcano plot presenting log2(fold change) vs log2(t test p). Each point represents a PC variable.

Data is presented for the discovery and validation TBI samples.

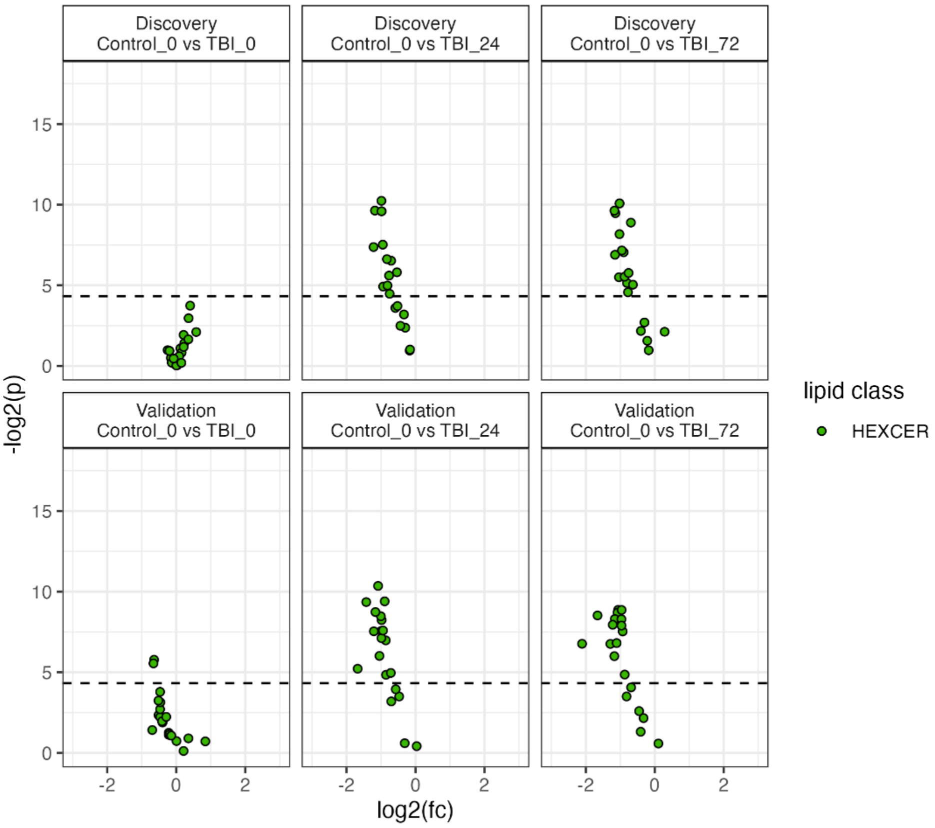

Supplementary figure S5: HexCer volcano plots

Volcano plot presenting log2(fold change) vs log2(t test p). Each point represents a HexCer variable. Data is presented for the discovery and validation TBI samples.

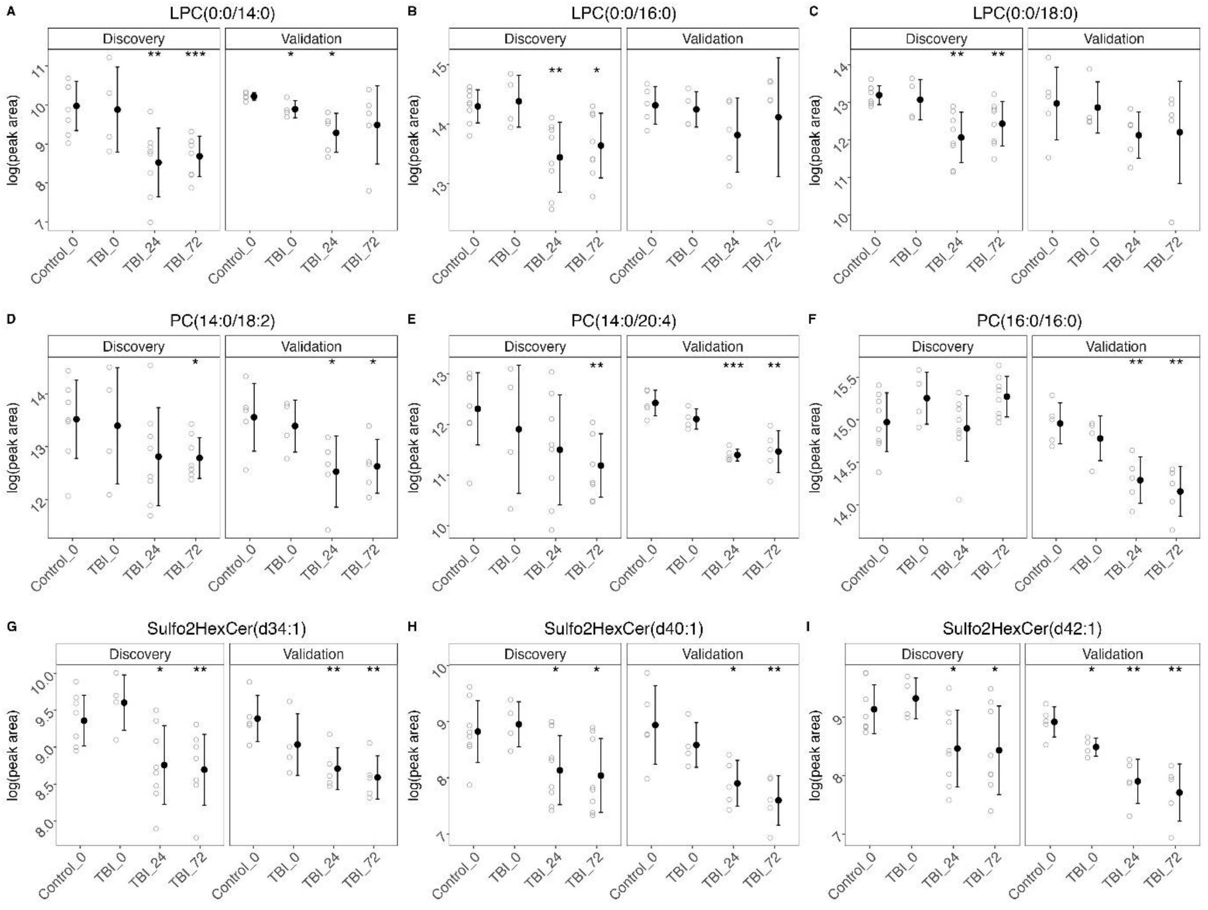

Supplementary figure S6: Individual standard error plots for exemplar lipids from LPC, PC and HexCer classes

Standard error plots for exemplar lipid variables that reported p <0.05 in t test analysis between control and a TBI time point in both the discovery and validation cohorts. Peak area values (y axis) underwent median factor normalisation between each batch.

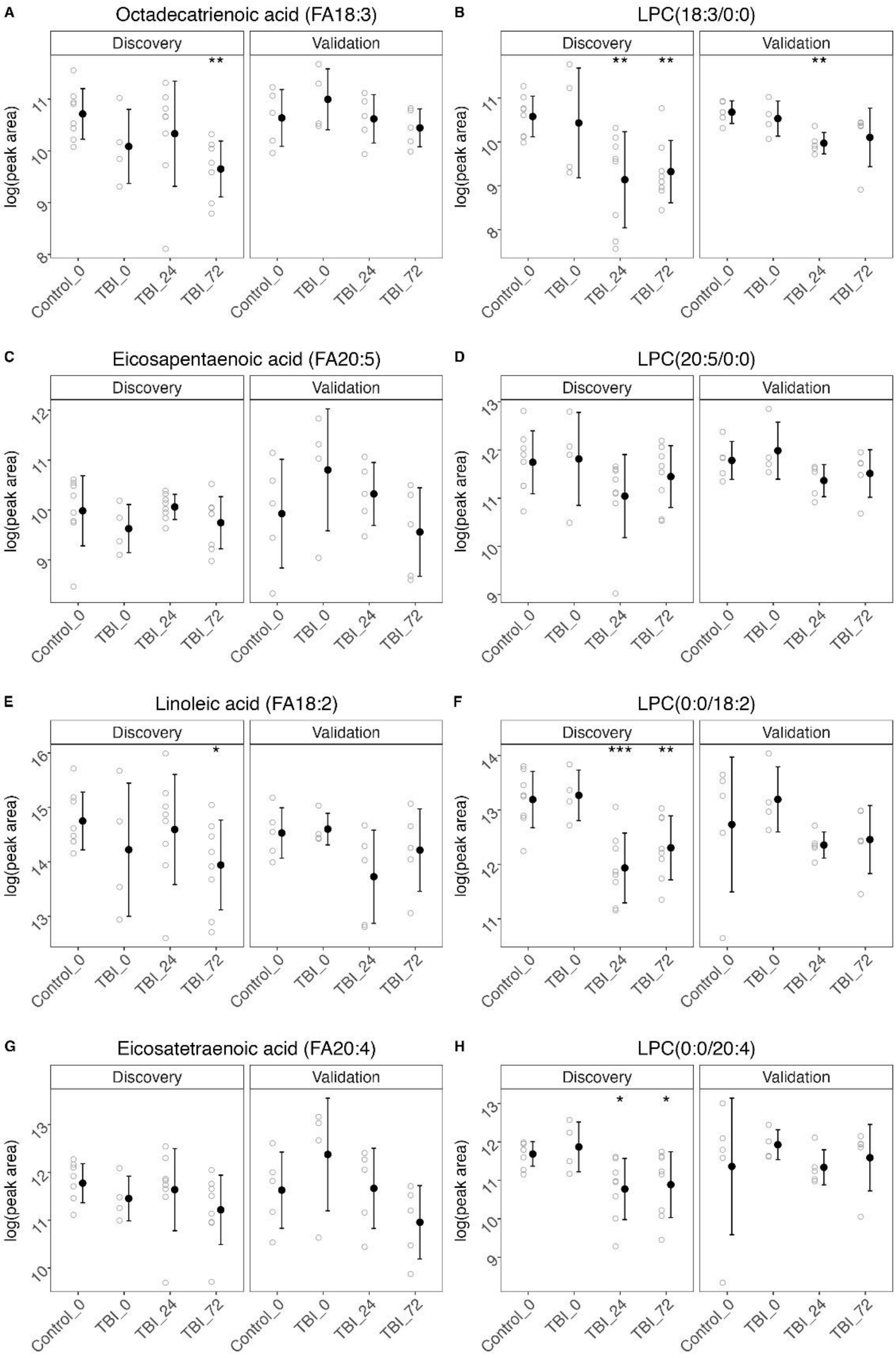

Supplementary figure S7: Exemplar omega-3 and omega-6 fatty acid variables

Standard error plots for exemplar omega-3 and omega-6 fatty acid variables in both the discovery and validation cohorts.

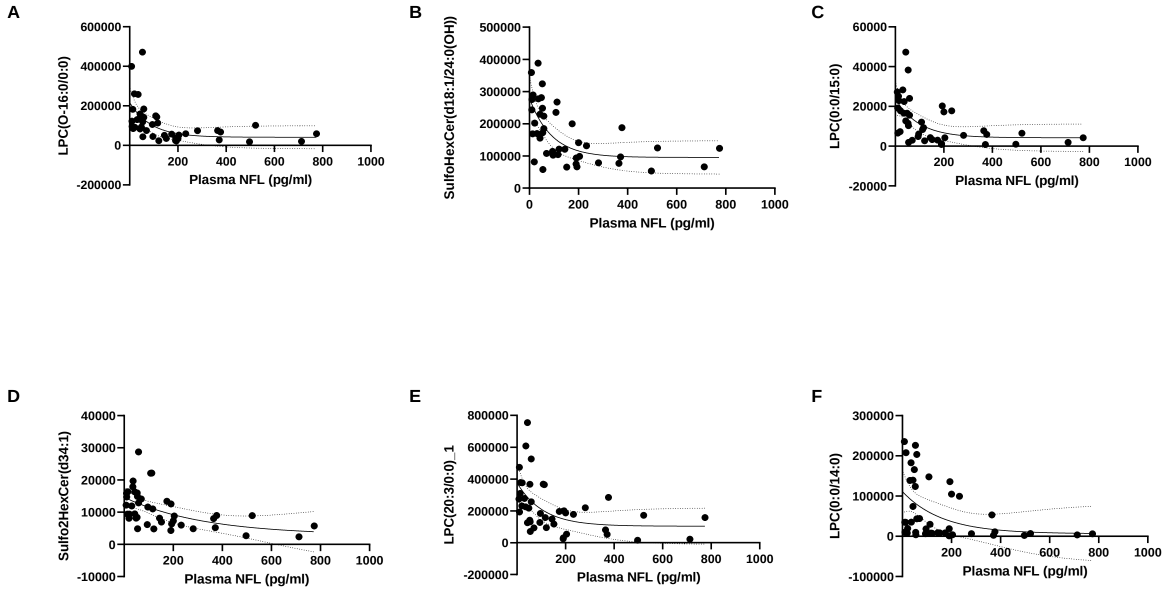

Supplementary figure S8: Top 6 lipids correlations with NFL

Plots of the top 6 negative correlations of LPC/LPE/HexCer/PC species with NFL. All correlations were significant (p < 0.001). Spearman correlation coefficient r for (A) LPC (O-16:0/0:0) = - 0.6781; (B) SulfoHexCer(d18:1/24:0(OH)) = -0.6226; (C) LPC (0:0/15:0) = -0.6217; (D) Sulfo2HexCer(d34:1)= -0.5738; (E) LPC(20:3/0:0)_1 = -0.569; (F) LPC(0:0/14:0) = -0.5388

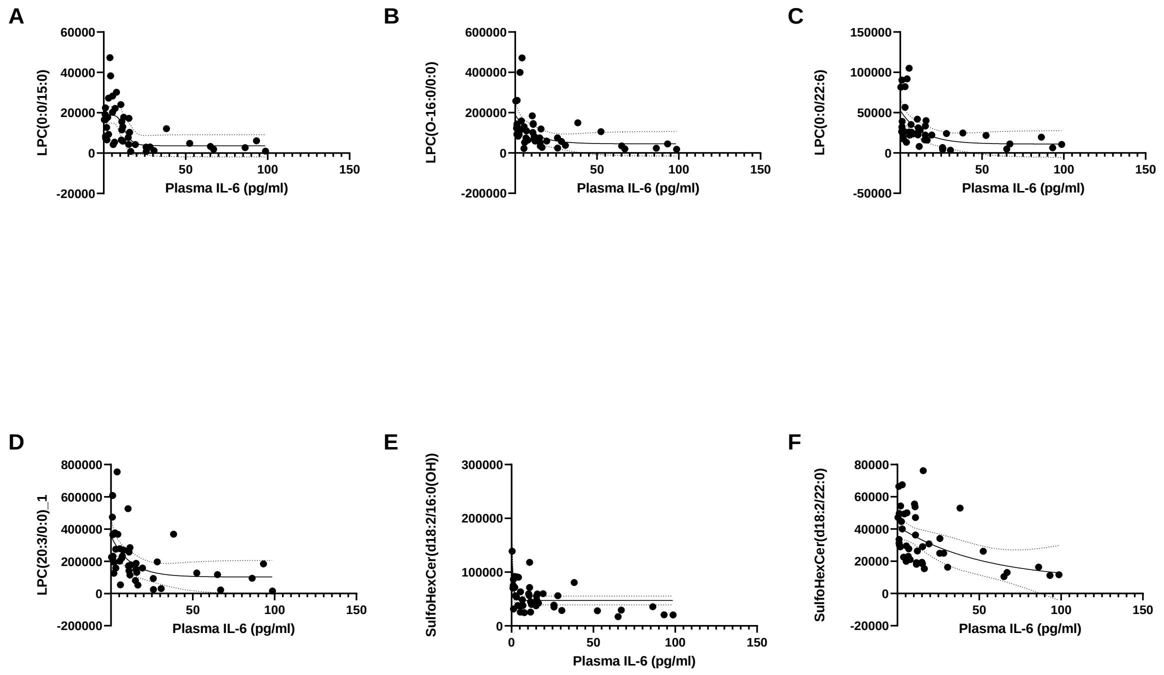

Supplementary figure S9: Top 6 lipids correlations with IL-6

Plots of the top 6 negative correlations of LPC/LPE/HexCer/PC species with IL-6. All correlations were significant (p < 0.001). Spearman correlation coefficient r for (A) LPC (0:0/15:0) = - 0.6477; (B) LPC (O-16:0/0:0) = -0.6262; (C) LPC (0:0/22:6) = -0.6213; (D) LPC (20:3/0:0)_1 = -0.6151; (E) SulfoHexCer(d18:2/16:0(OH))= -0.5726; (F) SulfoHexCer(d18:2/22:0)= -0.5667
